# Supplementary material for: Understanding reasons for suboptimal tuberculosis screening in a low-resource setting: A mixed-methods study in the Kingdom of Lesotho
Source: PLOS Glob Public Health. 2022 Mar 16;2(3):e0000249. doi: 10.1371/journal.pgph.0000249 (PMC10021370; doi:10.1371/journal.pgph.0000249)
Supplement: S1 Text — (DOCX) [file pgph.0000249.s001.docx]

**Semi-Structured FGD Guide for Nurses**

I want first to thank you for taking the time to be part of the focus group discussion today. My name ……………., and I am Dr. Afom Andom’s research assistant. We would like to discuss with you and your colleague's nurses about tuberculosis care services in your health facility. Specifically, We want to understand your thoughts and experiences about how patients move through the tuberculosis care cascade in your health facility. Your point of view as a health care provider is very important, so please feel free to share your opinions, experiences, and thoughts about TB services in the group discussion.

We want to remind you that whatever you tell us will be confidential and will not be shared with anyone outside of the research team. You are free to stop as at any time during the group discussions, and you have the right to skip any question that you do not feel comfortable with. Do you have any questions for me before we begin?

1. Tell me the role that nurses play in TB detection in your area?

2. Can you describe the challenges of TB detection in your health facility?

3. Can you tell me the effect of a lack of TB diagnostic capacity in the health centers on TB patients?

4. What are the effects of not being able to diagnose TB in health centers?

5. How frequent do you send samples from the health center to the district lab?

6. How regular do you perform the control sputum sample in your health facility? Tell me more?

7. How does the community view TB?

• Follow up: How does this affect your patients?

• Probe: Tell me more?

8. Can you tell me the workload at TB service in your health center?

Probe? What could be the reason?

9. Can you tell me how you feel when you inform patients that the result is TB positive?

10. Can you tell me the effect of TB on the life of the patients and their families?

11. Is there anything that you want to add on what you have told me?

**Semi-Structured Interview Guide for CHWs**

I want first to thank you for taking the time to speak with me today. My name ……………., and I am Dr. Afom Andom’s research assistant. Specifically, I want to understand your thoughts and experiences about how patients move through the tuberculosis care cascade in your health facility. Your point of view as a community health worker is very important, so please feel free to share your opinions, experiences, and thoughts about TB services.

I want to remind you that whatever you tell me will be confidential and will not be shared with anyone outside of the research team. You are free to stop me at any time during the interview, and you have the right to skip any question that you do not feel comfortable with. Do you have any questions for me before we begin?

1. How long have you worked as a community health worker in Berea hospital/Khubetsoana health center?

2. Tell me, in your capacity as a CHW, how do you help TB patients?

3. How regularly do you get training on tuberculosis?

4. Can you describe some of your job descriptions concerning TB?

5. Can you tell me how regular did you get supervision/ mentorship?

6. Thinking about the communities that you serve – how do they view tuberculosis?

Probes: a. Can you give me an example?

7. Can you describe why some tuberculosis patients come to the health facility very late?

Follow up: a. How about those who manage to come on time?

8. What helps patients to complete their medication?

9. What makes other patients of yours stop their medication?

Probe for an example.

10. Do you ever see patients coming to the TB facility early in the disease?

Probe: a. Tell me about these patients who come in early?

11. Do you ever see patients coming into the TB facility late in the disease?

Probe: Tell me more about these patients?

Follow up: a. What do you think the difference could be?

12. What the main challenges that you experienced at your work?

13. What keeps you motivated in your work?

14. What do you think should be done to improve TB detection?

15. Is there anything else that I should know to understand your experiences in TB care?

**Semi-Structured Interview Guide for District Health Manager**

I want first to thank you for taking the time to speak with me today. My name ……………., and I am Dr. Afom Andom’s research assistant. Specifically, I want to understand your thoughts and experiences about how patients move through the tuberculosis care cascade in your health facilities in Berea district. Your point of view as a district health manager is very important, so please feel free to share your opinions, experiences, and thoughts about TB services.

I want to remind you that whatever you tell me will be confidential and will not be shared with anyone outside of the research team. You are free to stop me at any time during the interview, and you have the right to skip any question that you do not feel comfortable with. Do you have any questions for me before we begin?

1. Can you describe for me your role in TB services as District Health Manager?

2. What could be the reason that health centers are not conducting TB diagnostic services?

Probe: a. Can you explain more?

3. Can you tell me the effect of not conducting TB diagnostic services to the patients and the population in the district?

Probe: Tell me more?

4. How do you send samples from the health facility to the district hospital?

Follow up: What works well about that system?

Follow up: What does not work well about that system?

5. How do you address the issue of sample storage?

6. How did you collect the samples from patients?

7. How do you transport samples from the health centers to the district hospital?

8. Who helps in transporting samples?

9. How is staff retention in your health facility?

Follow up: What could be the reason?

10. Can you tell me if there is a plan to start diagnostic services in the health facility?

Follow up: If yes, can you tell me more? If no, why not?

11. Can you tell me about the funding available for the TB program?

12. What are its effects on the implementation of TB programs in your district?

13. Tell me, how is the procurement process of TB drugs and lab reagent for TB?

Follow up: From where did you get your supply?

14. What do you think the main barriers to TB detection in your district?

15. What do you think should be done to increase TB detection?

(Probe to elicit multiple responses, then probe each)

16. Is there anything more to add to what you’ve told me? Do you have any questions for me?

**Semi-Structured Interview Guide for District TB Coordinator**

I want first to thank you for taking the time to speak with me today. My name ……………., and I am Dr. Afom Andom’s research assistant. Specifically, I want to understand your thoughts and experiences about how patients move through the tuberculosis care cascade in your health facilities in Berea district. Your point of view as a distric TB coordinator is very important, so please feel free to share your opinions, experiences, and thoughts about TB services.

I want to remind you that whatever you tell me will be confidential and will not be shared with anyone outside of the research team. You are free to stop me at any time during the interview, and you have the right to skip any question that you do not feel comfortable with. Do you have any questions for me before we begin?

1. Can you tell me about the diagnostic capacity of health facilities in your district?

Follow up: a. What do you think the strength of your diagnostic system?

b. What do you think the weakness of your diagnostic system?

2. Tell me the distribution of TB diagnostics in the health facilities?

Follow up: a. Hospital vs. Health center?

b. What do you think the reason is?

c. How does it affect patients?

3. What are the effects/burdens of TB on the lives of patients and their families?

4. How do you describe the effects of the lack of diagnostic capacity in the health centers in TB detection in the district?

Follow up: a. What do you think should be done?

5. Tell me the distribution of health care workers in the health facilities?

Follow up: a. How is the workload?

b. How is staff retention?

6. Can you tell me about the supervision and support for health care workers in the district in the TB program?

Follow up: a. How frequent is the support in a quarter?

7. Can you tell me the training program for the health care workers in the district?

Follow up: a. How frequent do you provide training?

8. How do you describe the TB data quality/documentation in your district?

Probe: can you tell me more?

9. What does the data flow look like in your district?

Probe: a. How is the data collection process?

b. How is data storage?

c. How is data usage. ?

10. Can you tell me the process of supply requisition and delivery of TB medications and lab reagents in the district?

Follow up: a. How effective is it?

11. What are the main TB detection challenges in your district?

Probe: a. Tell me more?

b. Do you have any example you can share?

12. What do you think should be done to increase the TB detection in your facilities?

a. Probe to elicit multiple responses, then probe each

13. Is there anything more to add to what you’ve told me? Do you have any questions for me?

**Semi-Structured Interview Guide for Implementing Partners**

I want first to thank you for taking the time to speak with me today. My name ……………., and I am Dr. Afom Andom’s research assistant. Specifically, I want to understand your thoughts and experiences about how patients move through the tuberculosis care cascade in the health facilities. Your point of view as a health implementing partner in TB program is very important, so please feel free to share your opinions, experiences, and thoughts about TB services.

I want to remind you that whatever you tell me will be confidential and will not be shared with anyone outside of the research team. You are free to stop me at any time during the interview, and you have the right to skip any question that you do not feel comfortable with. Do you have any questions for me before we begin?

1. Can you tell me about the background of your organization?

Probe: Tell me more?

2. What kind of support are you providing to the district in the TB program?

3. Can you tell me about the progress of your support in TB detection in Berera district?

4. Which area of your project went well?

5. What did not go well?

6. Can you tell me what were the main challenges of TB detection in the district are?

7. What do you think the effect of no diagnostic tool in the health centers on TB patients?

8. Can you tell me the issue of adherence to TB medication to patients?

9. Can you tell me about the district TB partners coordination? Probe: Tell me more?

10. Can you tell me how frequent did you meet with the TB partners and MOH to evaluate the progress of the TB program in the district?

11. How is the coordination in budgeting and planning between MOH and TB partners in the district? Probe: Tell me more?

12. From your experiences, how did you find working with the MOH?

Follow up: a. What were the strengths?

b. What was the weakness?

13. How do you explain the role of NGOs in TB detection?

Follow up: a. What did the performance of TB implementing partners look like?

Probe: a. Tell me more

b. What do you think the reason could be?

14. Can you tell me what can be done to improve TB detection in the district?

15. Is there anything more you want to add to what you have told me?

**Semi-Structured Interview Guide for Lab Personnel**

I want first to thank you for taking the time to speak with me today. My name ……………., and I am Dr. Afom Andom’s research assistant, and I would like to talk to you about tuberculosis care services. Specifically, I want to understand your thoughts and experiences about the services that you provided to TB patients in your Laboratory. Your point of view as a health care provider is very important, so please feel free to share your opinions, experiences, and thoughts about TB services.

I want to remind you that whatever you tell me will be confidential and will not be shared with anyone outside of the research team. You are free to stop me at any time during the interview, and you have the right to skip any question that you do not feel comfortable with. Do you have any questions for me before we begin?

1. What does a typical day in the lab look like for you?

2. Can you describe for me the laboratory services that your facility offers to TB patients?

Probe: Tell me more?

3. How often do you receive training in TB care services?

Follow up: a. What topics do you receive in training?

b. Who provides the training?)

4. How is the availability of diagnostic tests for TB?

5. How are the types of diagnostic tests for TB in your health lab?

Follow up: a. Tell me more about the distribution of diagnostic tests in the health facilities?

6. Tell me, has there ever been a time when the machines didn’t work?

Follow up: a. What was that like for you?

b. What do you do to make sure that the machines are functional?

c. What did that mean for patients?

6. Tell me about the staff that works on TB diagnostic tests?

Probe: Tell me more about human resources?

7. How is staff retention in your health facility? What could be the reason?

8. How regular is the stock of the supply of reagents for the laboratory?

Probe: tell me more?

9. Can you tell me the process of laboratory supply requisition process?

Follow up: a. How efficient is it?

10. If the sample does not process at the health facility, can you describe the process for testing samples?

Follow up: a. Where do you send the patients?

b. How far are the next facilities that patients can get tests?

c. How often do you send samples to the laboratory?

d. Where do you store the sample until you transport to the next level of the lab facility?

e. How do you transport the samples from the health facilities to the hospital laboratory?

11. Could you tell me about the sample rejection rate in your laboratory?

Follow up: a. what are the main reasons?

Probe: a. Tell me more about that?

12. Can you tell me about the result of the turnaround time?

Follow up: a. Tell me about a fast around time from your experiences

b. Tell me about the slow turnaround time?

c. What do you think the problem is?

13. How regular did the health facility send a sample test for control of the treatment at 2,3,5, and end of treatment?

Probe: a. can you tell me more?

14. How do you communicate the result with patients?

15. What are the mains challenges that you have in your laboratory?

Follow up: a. What does that challenge mean for your work?

b. What does that challenge mean for patients?).

16. What could help overcome that challenge?

17. What could be done to improve TB diagnostics, from your point of view?

18. Is there anything you want to ask me?

**Semi-Structured Interview Guide for TB Screeners**

I want first to thank you for taking the time to speak with me today. My name ……………., and I am Dr. Afom Andom’s research assistant, and I would like to talk to you about tuberculosis care services. Specifically, I want to understand your thoughts and experiences about how patients move through the tuberculosis care cascade in your health facility. Your point of view as a health care provider is very important, so please feel free to share your opinions, experiences, and thoughts about TB services.

I want to remind you that whatever you tell me will be confidential and will not be shared with anyone outside of the research team. You are free to stop me at any time during the interview, and you have the right to skip any question that you do not feel comfortable with. Do you have any questions for me before we begin?

1. Can you tell me how sick the patients are that you were screening? How do you know?

2. What are the main barriers of patients to access TB screening services in your health center?

Probe: Tell me more? Probe for an exhaustive list of barriers.

3. Can you tell me the effect of TB on the life of TB patients? Tell me more?

4. What role do health workers play in getting patients to come to health facilities?

5. Can you tell me the perceptions of families and communities on TB?

Follow up: How does that affect your patients?

Probe: Can you share it with me if you have an example?

6. Can you tell me the process of TB screening in your health facility?

7. How frequent do you collect a sputum test?

8. How do you transport sputum samples?

9. Where do you store it?

10. What do you think should be done to improve tuberculosis screening in your facility?

Probe to elicit multiple responses, then probe each

11. Is there anything more to add to what you’ve told me?

12. Do you have any questions for me?

**Semi-Structured Interview Guide for Patients at the Screening Point**

I want first to thank you for taking the time to speak with me today. My name ……………. I am Dr. Afom Andoms's research assistant. I would like to talk to you about tuberculosis care services. Specifically, I want to understand your thoughts and experiences as a patient on the TB services that you have receive in this healh facility. Your point of view as a health care provider is very important, so please feel free to share your opinions, experiences, and thoughts about TB services.

I want to remind you that whatever you tell me will be confidential and will not be shared with anyone outside of the research team. You are free to stop me at any time during the interview, and you have the right to skip any question that you do not feel comfortable with. Do you have any questions for me before we begin?

1. Can you walk me through the processes of your illness?

Probe: When do the symptoms start?

2. How many times have you visited the health facilities for this problem?

Probe: Why that many times?

3. Can you tell me if you had any previous history of tuberculosis?

Follow up: Do any of your friends or family members have an active cough for more than three weeks or had one in the past year?

Probe: a. Is there any sick person at home?

4. How do you prepared to come to the health facility?

5. How did you get to the facility today? Please walk me through it.

Probe: a. How far is your home from the health facility?

b. How many hours did you walk/travel?

c. What is the condition of the road

d. What type of transport did you use?

e. How much did you pay?

6. How would you describe the TB services that you received at the health facility?

Probe: a. Please walk me through your whole day starting when you arrived at the health facility?

7. How did the staff at the facility treat you?

Probe: a. How did that make you feel?

b. Can you give me an example?

8. Can you tell me your feeling when the health worker told you that you would get investigated for TB?

Probe: a. Can you tell me more about that?

9. What directions were you given about how to collect your sputum?

Follow up: a. Who gave you the directions?

b. What was the content of the messages?

10. What are your plans for the next steps?

Follow up: a.What will help you take that next step?

b. What will make it hard to take that next step?

11. Can you tell me the attitude of your family, friends, and community on TB?

Follow up: How do they explain to TB?

12. How have your symptoms affected your life?

Follow up: How have they affected your family?

13. What would have made it easier for you to get this far in care?

14. What could be done better for others in your situation?

15. Is there anything else important you would like to share with me?

**Semi-Structured Interview Guide for Patients who Had Sputum Samples Sent to the Lab**

I want first to thank you for taking the time to speak with me today. My name ……………., and I am Dr. Afom Andom’s research assistant. I would like to talk to you about tuberculosis care services. Specifically, I want to understand your thoughts and experiences as a patient on the TB services that you have receive in this health facility. Your point of view as a patient is very important, so please feel free to share your opinions, experiences, and thoughts about TB services.

I want to remind you that whatever you tell me will be confidential and will not be shared with anyone outside of the research team. You are free to stop me at any time during the interview, and you have the right to skip any question that you do not feel comfortable with. Do you have any questions for me before we begin?

1. What was it like for you to get to the facility?

Follow up: a. How did you prepare to come to the facility?

2. What needs to be taken care of for you to be able to return to the facility?

3. When you gave the sample, what did the health care worker tell you?

Probe: a. Tell me the plan for you to pick up the results?

b. What did you think about that?

c. You have made it through lots of steps so far, what keeps you coming back?

4. First, you had sputum collection, what was that like for you?

Follow up: a. How about when you had to come back for the sample?

b. How did that go?

5. Tell me how long it will be until you get the result?

Follow up: a. In your view, why will it take that long?

6. What did you do while you were waiting for the lab result?

7. What are the effects/burden of TB on your life?

8. What is the effect of TB on the life of your family?

Probe: a. Tell me more about it?

b. Can you share it with me in case you have an example?

9. Can you tell me what the perception of your family, friends, and your community on TB disease is?

Probe: a. What do you think the reason could be?

10. What problems have you experienced during your illness?

Probe: a. Tell me more?

11. Is there anything else you want to share with me?

**Semi-Structured Interview Guide for Patients who Started Anti-TB Treatment**

I want first to thank you for taking the time to speak with me today. My name is………….. I am Dr. Afom Andom's research assistant, and I would like to talk to you about tuberculosis care services. Specifically, I want to understand your thoughts and experiences as a patient on the TB services that you have received in this health facility. Your point of view as a patient is very important, so please feel free to share your opinions, experiences, and thoughts about TB services.

I want to remind you that whatever you tell me will be confidential and will not be shared with anyone outside of the research team. You are free to stop me at any time during the interview, and you have the right to skip any question that you do not feel comfortable with. Do you have any questions for me before we begin?

1. When did your symptoms start?

2. How did you prepare to come to the health facility?

3. How many times have you visited the health facilities? For what purpose?

4. Can you walk me through the processes of your illness?

5. How did you get to the facility today?

Follow up:

a. How far is your home from the facility

b. how long did you take to come to the facility?

c. How far is your home from the health facility?

d. How accessible is the road?

e. How is the availability of transport?

f. How much does it cost? How do you handle that?

6. How would you describe the tuberculosis services that you receive in the health facility?

Follow up:

a. Can you walk me through sample collection?

b. Tell me about how screening went for you?

c. Tell me how the lab test process was?

7. Can you tell me what was your feeling when you told you had a positive TB diagnosis?

Follow up: How did that go for you?

Probe: Can you tell me more?

8. What are the effects of TB on in your life and life of your family?

Probe: a. Tell me more?

b. Can you share it with me in case you have an example?

9. Tell me, what will your TB treatment look like for you?

Probe: a. why?

b. Tell me more?

10. What feels good to you about you starting treatment?

Probe: Tell me more?

11. What feels difficult for you about starting treatment?

Probe: Tell me more?

12. How do you describe the support of your family, friends, and community during your illness?

Probe: a. Tell me more?

b. Can you give me an example?

13. Can you tell me about the coping mechanism that you were using to overcome some of the challenging experiences of your illness?

Probe: a. How was it?

b. Can you tell me more?

14. You have seen a lot of health care workers during this time.

Follow up: a. Tell me, who has been helpful?

b. How did they help you?

c. In what way have the providers not been helpful?

d. What was that like for you?

15. How did you find the attitude of the health care workers during the courses of your illness?

Probe: tell me more?

16. You have come a long way since your first visit to the clinic with your cough.

Follow up: a. What kept you coming back?

b. What was hardest about coming back for the next step?

c. Yet you still managed to come back. How did you do it?

17. Is there anything else important you would like to talk about?

**Semi-Structured Interview Guide for TB patients after TB Treatment Completion**

I want first to thank you for taking the time to speak with me today. My name………………. I am Dr. Afom Andom's research assistant. I would like to talk to you about tuberculosis care services. Specifically, I want to understand your thoughts and experiences as a patients on the TB services that you have receive in this healh facility. Your point of view as a health care provider is very important, so please feel free to share your opinions, experiences, and thoughts about TB services.

I want to remind you that whatever you tell me will be confidential and will not be shared with anyone outside of the research team. You are free to stop me at any time during the interview, and you have the right to skip any question that you do not feel comfortable with. Do you have any questions for me before we begin?

1. What was it like to get to the facility?

Follow up: a. How did you prepare for the day?

b. What needs to be taken care of for you to be able to return to the facility?

2. How did you prepare to come to the facility?

3. Can you tell me, how do you feel after you completed the whole course of TB treatment?

Probe: What makes you feel that way?

4. What did you think of the treatment when you first started?

Follow up: Did your thoughts or feelings about treatment change in any way as time went on? Probe: Tell me about that.

5. I want to learn more about how your life has changed before, during, and after TB treatment. Let's start with before.

Follow up: How about during your treatment?

Follow up: And now that you have completed treatment

6. Can you tell me what the effects of TB on your life and the life your family was?

Probe: Tell me more?

7. Can you describe your relationship with your family and neighborhood after you diagnosed with TB?

Follow up: How about when you were undergoing treatment?

Probe: tell me more?

8. How did you describe the contribution of your family and friends during the courses of your illness?

Probe: tell me more?

9. How did you describe the contribution of health care workers during your illness?

Probe: Tell me more?

10. What were the main challenges with taking your treatment?

Follow up: a. What made that so difficult?

b. How did you cope with that challenge?

11. What problems have you experienced in the course of your illness?

12. What do you think should be done to improve TB services?

13. Is there anything else important to you would like to talk about?
