# Supplementary material for: Understanding reasons for suboptimal tuberculosis screening in a low-resource setting: A mixed-methods study in the Kingdom of Lesotho
Source: PLOS Glob Public Health. 2022 Mar 16;2(3):e0000249. doi: 10.1371/journal.pgph.0000249 (PMC10021370; doi:10.1371/journal.pgph.0000249)
Supplement: Attachment — Submitted filename: Reviewer response.docx [file pgph.0000249.s003.docx]

Thank you to the reviewers for their thoughtful comments on our manuscript. We have substantially revised the paper, refocusing it on TB screening (as suggested by reviewer 1), and we believe it is much stronger. Point-by-point responses to specific comments are below.

Reviewer #1: 

**Comment 1:** First, and perhaps most importantly, the paper is really an exploration as to why the rates for TB SCREENING are low, not as to why the rates of TB detection are low. While it is great that the authors present all steps of the cascade from the quantitative analysis, the fact that they only explore the reasons behind low rates of screening in the qualitative assessment really means their paper should focus on this aspect of the cascade. Thus I would suggest that they reframe the entire paper in this way.

a) This should start with the title--which should be changed to state it is TB SCREENING and not DETECTION that will be explored--and carry throughout the paper. I think this might help the authors focus the background and discussion more as well.
b) And while I think it is worth presenting the whole cascade with its results, the authors really should try and target the paper more on TB screening.
c) To this end, it would help to have something in the background about the health care resources/services available in Lesotho as well as other experiences with implementing TB symptom screening systematically (for example this has been done in South Africa for quite some time).
d) I also think it would be important in the discussion section to talk about the limitations of TB symptom screening as part of the care cascade and how it misses many people with active TB, even when done systematically. I find it interesting, for example, that the authors note in their own introduction section in lines 41-43 that screening should preferably done with chest X-ray, thus suggesting they also see a limit to TB symptom screening. But since the bulk of the paper is about why TB symptom screening is not done, I would suggest the authors substantially revise the paper to focus on this. I suspect this will take a couple of rounds of revisions with reviewers, but I would encourage the authors to try and do this if they are willing as there are some rich and important findings in their study.
e) If the authors want to focus on other aspects of the care cascade, then there are some other findings that merit discussion that are completely unaddressed in their mixed methods. Their rates of sputum collection and submission are strikingly high as are their rates of treatment initiation, and this should be commented on if the authors chose to explore other aspects of the cascade. However, their rates of treatment completion are unacceptably low and this should also be explored. It was not clear to me if authors actually collected qualitative data on these aspects of the cascade and simply chose not to present them for reasons of space/economy or of the only collected qualitative data on why people did not undergo screening. If they have qualitative data on these other aspects of the cascade, they could certainly consider presenting them in a revised manuscript--although I suspect it could get quite lengthy.

**Response**: Thank you to the reviewer for this thoughtful set of suggestions – we have divided them up into sub-points to help make our response easier to follow. To explain the situation: we collected both quantitative and qualitative data on the entire cascade, but the volume of qualitative data was so large that it was not feasible to include it all in a single article. We therefore chose to focus on screening, which was the step of the cascade where the largest gap was observed. However, we agree that this created a mismatch between the quantitative and the qualitative data and leads to confusion about the scope of the article. Therefore, we have taken the reviewer’s first suggestion to refocus the paper on screening. Specifically:

a) We have changed the title to “Understanding reasons for suboptimal tuberculosis screening in a low-resource setting: a mixed-methods study in the Kingdom of Lesotho”
b) We have removed the reporting of the full care cascade and only reported the results of the symptom screening in the quantitative data section. The quantitative and qualitative data therefore now match in scope. We have also removed the paragraph in the discussion that focuses on care cascade evaluation and added paragraphs (described in some of the responses below) that discuss additional findings related to screening.
c) We have substantially revised the introduction to focus on TB screening. We have revised the initial paragraph to focus on the rationale for screening people in health facilities and the evidence around this practice in other countries, adding the following text:

“In settings with high TB burdens, actively screening key populations for TB is a critical step for closing the case detection gap [4]. In settings with high TB burdens, one population that can benefit from systematic TB screening is people attending health facilities. In settings with high HIV prevalence, health facility attendees include large numbers of people living with HIV, making facility-based screening particularly effective for TB detection. Prevalence surveys in sub-Saharan African countries have repeatedly found that many people with undiagnosed TB had in fact sought care for their symptoms [5]. Screening health facility attendees has been shown to increase overall TB diagnoses in the population in countries with high TB and HIV burdens [6,7]. However, even though countries may have policies for screening health facility attendees, incomplete implementation leads to missed diagnoses [8,9].”

d) We have added the following paragraph to the discussion about the limitations of symptom screening and screening within health facilities, and what more is needed:

“While symptom-based screening in health facilities is potentially low-cost and high-yield, it is likely not sufficient to close the gap in case detection in Lesotho or other similar settings. Known barriers to TB case detection in Lesotho include poor access to health care in rural areas [22] and suboptimal logistics for sputum testing [23. Thus, efforts to improve case detection cannot be limited to health facilities. Bringing active case-finding services into communities can help to improve case detection among people who face barriers to accessing health facilities [24]. Indeed, over half of the people with respiratory symptoms identified by the 2019 Lesotho TB prevalence survey had not sought care for their symptoms [25]. Moreover, symptom screening has serious limitations, as prolonged cough has poor sensitivity while broad symptom criteria have poor specificity [4]. Chest radiography offers high sensitivity and specificity as a screening tool, and computer-aided detection software can help to make mass radiographic screening feasible [26]. In settings with high TB burdens, community-based mobile radiography units have been used to reach populations that face barriers to accessing health facilities [27] and have been shown to increase population-level case notifications [28].”

e) We agree that presenting all the qualitative findings for the full care cascade would make the paper too long. We have chosen to take the reviewer’s first suggestion of limiting the paper to TB screening.

**Comment 2**: My second major concern with the paper is that there seem to be some internal inconsistencies with the data shared in the qualitative assessments and the data in the quantitative cascade. This happens often in mixed methods research, as people are often inconsistent with reporting or understanding the motivations for their behaviors. But when this happens it must be addressed and explored in the discussion section. One example of this is the reported qualitative finding that nurses did not "trust" the screeners to really do the TB symptom screening and thus they re-screened the patients themselves. But if this is true--then it would mean that people were screened TWICE at the health facilities (once by screeners and once by nurses) and rather a higher rate of symptom screening should be reported (unless this is, of course, not really happening or not being "recorded" in a way that was captured in the data analysis).

**Response**: We have added the following sentences to a paragraph focusing on the limitations of the quantitative estimate of screening coverage:

“Our assessment of the number of people screened based on screening registers is also subject to error in both directions. On the one hand, people who were screened twice – once by a screener and once during their clinical evaluation – could potentially be listed on the screening register twice. However, in practice, nurses who screen patients during the clinical evaluation often do not fill out the register. While there is thus substantial uncertainty around our estimate of screening coverage, the challenges revealed by our qualitative findings suggest that even if the registers underestimate the number of patients screened, the quality and consistency of screening is likely to be suboptimal.”

We have also touched on this issue in the discussion of possible models for improving TB screening, noting that, “Since health care workers indicated that TB screening within the context of the clinical evaluation sometimes occurs already, standardizing and monitoring the process could replace a separate screening procedure.”

**Comment 3**: Another example is saying that patients have to wait for long periods of time when they come to the clinic but that they do not have time to undergo TB screening. If they are queued and waiting for a long time, could the screening not also be done then? During the early days of antiretroviral availability in Lesotho, for example, the long waiting times were used to do HIV education and counseling, as people were queuing for hours to wait for physicians. While it may be that people did not want to talk about TB or get "pulled" from the queue to do further TB testing, this should lead to a discussion on the need to better streamline TB screening and testing procedures for people. There are other instances of this throughout the paper that must be addressed in the discussion and limitations section, as some of what people appear to be reporting does not match what the numbers show. This is one of the beautiful things about mixed methods studies in that you have different information coming from different actors/sources. But these things must be discussed.

**Response**: Currently, patients are being screened as they are queued and waiting for care, as indicated by the guidelines. However, the problem is that if they report symptoms, they will be asked to go to the TB clinic to give sputum as a separate activity from whatever care they are at the health facility to receive, prolonging the total time spent at the health facility. We have clarified this in the results:

“Health care workers expressed concern that, in their view, patients provided answers to a screening question that would not prolong their time at the clinic by requiring them to visit the TB clinic for sputum collection.”

We have also incorporated this issue into the discussion of two possible models for improving TB screening (see full paragraphs in response to Comment 8):

- If screening is conducted outside the clinical consultation by layperson or community health worker screeners, “it would be necessary to integrate TB screening efficiently into the patients’ visits so that sputum collection does not prolong the time that patients spend at the clinic.”
- If screening is integrated into standard intake or clinical evaluation procedures rather than being a separate activity, “Making screening and sputum collection part of a standard intake or evaluation procedure could potentially also reduce the disincentive for patients to respond positively to TB screening if it reduces the perception of TB screening as an additional activity that prolongs the time spent at the health facility.”

**Comment 4:** There is a different in the percentage of people screened for TB symptoms at the hospital versus the health center and the authors should comment on this in the discussion and present any qualitative data they might have that could illuminate the difference. One would expect a hospital to be better staffed and resourced than a health center--although perhaps they were also overwhelmed with too many critically ill patients? Whatever the reason, this should be noted and explored.

**Response:** We went back through all the qualitative data to see if there was anything that might shed light on this difference but could not find anything. We have added a speculative explanation based on the personal experience of the authors: “We found that less than a third of visitors at the district referral hospital and around half of the visitors at the health center were asked about TB symptoms. While we do not know the reason for this difference, is possible that TB screening activities are less emphasized at the hospital based on the assumption is that patients would have already been screened at their local health center prior to referral to the hospital.”

**Comment 5:** The interview guide should be included as an Annex.

**Response**: We have uploaded the focus group discussion and interview guides as a supplement. Note that we have included the full guides even though this manuscript only deals with the portion of the qualitative data related to screening, as comments related to screening arose in response to different questions.

**Comment 6**: There should be a COREQ checklist used to report on this qualitative study and that should be submitted with the revision and noted in the methods.

**Response:** We have uploaded the COREQ checklist and noted its use in the methods. Page numbers in the checklist refer to the clean version of the manuscript.


**Comment 7**: Was there any attempt in the estimates of number of people who should be screened for TB to account or adjust for TB screening in children, where different symptoms might suggest TB? Where sputum may be difficult to obtain? This should be mentioned since the authors note they used data from the vaccine and under 5 clinics to calculate the number of people who should have been screened for TB.

**Response**: The district reports list the number of visits, not the number of individual people entering the health center. Because young children do not access health services independently, each visit to a child-focused health service such as vaccination or <5 clinics would involve a child accompanied by a guardian, who should be screened as an adult. We have added the following passage to the limitations:

“To estimate the total number of visits, we assumed that each visit corresponded to a single individual eligible for screening, even though for child-focused services, each visit would involve multiple people, as children would be accompanied by adult guardians. Since cough-based screening is an adult-focused strategy, we believe that using total visits as a denominator is valid, as the total number of visits is likely to be close to the total number of adults coming to the health facility. However, this assumption reflects the lack of systematic screening of children for TB.”

**Comment 8**: In line 278-279, the authors note that the "lack of specialization" may be one reason why TB screening was not done. I am not sure if this is their term of if this is what participants gave as a reason. It is worth exploring this further in the discussion as well, since one "goal" of a PHC is that everyone can do multiple jobs--and it is precisely this lack of "specialization" that is the goal in an integrated, holistic system. Screening for TB symptoms is not a complex intervention where "specialization" is needed. So how would this fit in with a whole UHC goal--which I know Lesotho has invested a lot in--as a way to improve TB care? Again, if this is what participants stated as a reason for not doing TB screening, then the authors should report it this way. But this is an important finding that then has implications for trying to get all PHC staff or community health workers to be able to do TB screening and shows the global move towards universal screening may not match with what people on the ground are seeing or want ("specialized people" to do screening).

**Response**: We have gone back to the qualitative data to try to refine our analysis of this complex issue. We have made the following revisions to the manuscript.

First, we have combined what were originally subthemes 1A (workforce not tailored to health facility needs) and 1B (staff covering multiple programs) under 1A. While multiple health care workers referred to the multiple programs and the inability of the staff to cover all the programs, we decided that this was best discussed as a specific example and consequence of staffing plans not being tailored to the needs of the health facilities. We have revised the description of this finding in the results:

“One consequence of understaffing is that all staff are expected to provide all forms of care, regardless of their training or affiliation with a given program. Under this work-sharing model, each staff member switches between programs on any given day to ensure that all activities in the facility are covered. The expectation that staff swap roles to meet care demands means that staff must often work without taking any breaks.”

Second, the term specialization was not used by the participants, so we have removed this from our interpretation. What was directly stated by the participants was a desire for people to be specifically trained on TB screening, and for it to be clear who was responsible for TB screening. In the discussion, we have added the following paragraphs on two potential solutions to this challenge based on existing models that have proven successful in other contexts. One is to have dedicated layperson or community health worker screeners – a form of specialized staff, but not the existing health facility staff. Another is to integrate TB screening into standard general intake or clinical evaluation procedures, which is more in line with the primary health care approach.

“Our findings showed that the overall shortage of staff and lack of trained TB screeners contributed to the low completion of TB screening. These factors have been found to be major barriers to effective TB screening in other settings as well, contributing to poor service quality, long health service delays, low patient satisfaction, and high staff turnover [15,16]. One potential solution to the lack of ownership over TB screening activities described by the health care workers in our study would be to introduce trained layperson or community health worker TB screeners into health facilities, which has been shown to increase TB case detection [6,17]. This would require additional resources, as screeners are likely to be more effective if properly compensated [18]; however, using community health workers for activities that do not require a nurse or doctor has been shown to be cost-effective across a variety of health conditions and interventions [19]. Moreover, it would be necessary to integrate TB screening efficiently into the patients’ visits so that sputum collection does not prolong the time that patients spend at the clinic.”

“Another potential strategy for improving TB screening in the context of understaffed health facilities is better integration of health services. In our study, health care workers mentioned that having insufficient staff to cover multiple vertical programs attending to different health conditions contributed to a lack of prioritization of TB screening – a separate activity not tied to the other programs. Integrating TB screening into standard health facility intake procedures could increase coverage with minimal additional time required [20]. Alternatively, TB screening could be incorporated as a standard evaluation procedure at every clinical encounter, as has been done successfully for people accessing HIV care in many sub-Saharan African settings [21]. Since health care workers indicated that TB screening within the context of the clinical evaluation sometimes occurs already, standardizing and monitoring the process could replace a separate screening procedure. Making screening and sputum collection part of a standard intake or evaluation procedure could potentially also reduce the disincentive for patients to respond positively to TB screening if it reduces the perception of TB screening as an additional activity that prolongs the time spent at the health facility.”

**Comment 9**: Some of the language needs to be more scientific and less informal. For example, the test done to look for TB is a cartridge-based genotypic test and not a "GeneXpert" machine (line 45), it is not clear what a "mild" symptom is (line 42). The group that transports the sputum specimens is called Riders for Health (not Riders on Health, line 82). There is also some stigmatizing language used in the paper that should be addressed (i.e. while it is a common epidemiology term to refer to "TB cases" these are people who are living with TB--line 62).

**Response**: We have gone through the manuscript to check and formalize the language. We have restricted the usage of the term “case” to the phrase “active case-finding” or the one instance where it was used in a direct quote (“we are missing TB cases”). We have removed all instances where “case” could have been construed as referring to a person rather than an instance of disease.

**Comment 10**: I realize I am suggesting a major re-write/reconceptualization of this piece of work, but I would encourage the authors to consider it if they can. I think since the bulk of the qualitative data are presented on why people are not doing screening, then the paper should be revised around that. If the authors do want to focus on the whole cascade, then they need to present qualitative data on the other aspects of the cascade, because some of the steps are completed at a higher rate than would be expected (sputum collection, initiation in treatment) and others are also much lower than expected (treatment completion).

**Response**: Thank you for all the thoughtful comments. We have decided to refocus the paper on screening only. After removing the text related to the cascade as a whole, we have used the space instead to dig deeper into the interpretation of the screening results; we hope that we have achieved a more thorough discussion of the qualitative and quantitative findings as suggested in comments 2-4 and 8 above. We will present the rest of the cascade in a separate paper, where we will have space to use the qualitative data on these other cascade steps to explain both high and low completion.

Reviewer #2:

**Comment 1**: Authors need to ensure consistency in the use of British and American, in the abstract the authors have used Supervised the British way and Incentivized the American way

**Response**: We have gone through the article and used American spelling conventions.

**Comment 2**: The conclusion of the study is not supported by the study findings. The authors have indicated in the conclusion that case finding could improve if health care workers are supervised. I did not see any data supporting this conclusion

**Response**: We have revised the conclusion paragraph. What we meant by “supervision” is better described as monitoring of TB screening activities to promote accountability, reflecting health care workers’ concerns that no one felt responsible for TB screening. Such monitoring would also make clear to the health facility the gap in screening coverage that our current study has observed. We have replaced the statement about supervision with the sentence

“Within health facilities, it is necessary to ensure that the staff conducting this activity are properly trained and that the coverage and quality of screening is continuously monitored.”

**Comment 3**: In the discussion, the authors need to relate their findings with others studies. They made an attempt but not consistently.

**Response**: We have revised the introduction to present information from additional studies about screening in health facilities (references 4-7 and 9), and we have incorporated additional studies into our interpretation of findings in the discussion (references 18-21, 26-28).
