# Supplementary material for: Understanding reasons for suboptimal tuberculosis screening in a low-resource setting: A mixed-methods study in the Kingdom of Lesotho
Source: PLOS Glob Public Health. 2022 Mar 16;2(3):e0000249. doi: 10.1371/journal.pgph.0000249 (PMC10021370; doi:10.1371/journal.pgph.0000249)
Supplement: Attachment — Submitted filename: Response to reviewers.docx [file pgph.0000249.s004.docx]

Thank you to the reviewers for their careful review of our revision. We have made the requested changes as described below.

**Reviewer 1**

**Comment 1:** I have one very minor comment to make. The authors use the term "visitors" to the health facilities, and I understand this to mean people who are coming there to seek health care. However, when referring to the hospital, the term visitors could also mean people who are there to visit family members or other people who are sick in the hospital. Are these people also screened for TB? Should they be? Or do the authors mean to use the term "visitors" to convey people who come there seeking care? I realize this is minor, but the term should probably be clarified somewhere in the paper (i.e. a small sentence saying "We use the term "visitor" to denote people who came to the facility seeking health services" if this is in fact what is meant. I do note that the authors state that family members often brought children for care and those family members are screened as well, so maybe they do mean the term to encompass anyone entering the facility for any reason, and if so, this should be mentioned.).

**Response:** We used the term visitor to mean people seeking services or the guardians accompanying children for care. We did not include people coming to visit hospital inpatients. We have added this clarification at line 100-102: “We calculated the percentage of people screened by dividing the number screened by the total estimated visits to the health facilities, assuming that each visit corresponded to a single visitor who should be screened (i.e. a person accessing health services for him/herself or a guardian bringing a child to access services).”

**Reviewer 2**

**Comment 1.** In the abstract they have used TB screeners, this is not an official term in reference to the health care workers involved in screening for TB. The author may consider using the term clinical team

**Response:** We have changed “trained tuberculosis screeners” to “training on tuberculosis screening” to avoid specifying a particular cadre of people.

**Comment 2:** In line 83 the term once found positive for TB symptoms, can be substituted with TB presumptive

**Response:** We have removed the redundancy in this sentence by deleting the initial clauses so that it states simply, “All those with TB are initiated on treatment, which is available for free throughout the country.”

**Comment 3.** In line 108 - 112 the authors need to observe the space before the = sign

**Response:** We have added spaces

**Comment 4.** in line 477 I suggest that for the reference 29 and 30 they use close the square brackets for each reference as [29], [30]

**Response:** We have checked a couple of articles from this journal and found that when two references are cited, they are enclosed in a single set of brackets. We have therefore retained this formatting throughout.
